# Effect of Electron Beam Irradiation on the Percentage Loss of Tensile Modulus of Epoxy Polymer

**DOI:** 10.3390/polym17040447

**Published:** 2025-02-08

**Authors:** Lingzhi Cong, Zhibin Guo, Xin Zhang, Huyang Li, Hao Jiang, Yuhang Jing, Jihong Yan, Weiqi Li, Jianqun Yang, Xingji Li

**Affiliations:** 1 Department of Astronautical Science and Mechanics, Harbin Institute of Technology, Harbin 150001, China; 2 State Key Laboratory of Robotics and Systems, Harbin Institute of Technology, Harbin 150001, China; 3 Technology Innovation Center of Materials and Devices at Extreme Environment, Harbin Institute of Technology, Harbin 150001, Chinalxj0218@hit.edu.cn (X.L.); 4 Laboratory for Space Environment and Physical Sciences, Harbin Institute of Technology, Harbin 150001, China; 5 School of Physics, Harbin Institute of Technology, Harbin 150001, China

**Keywords:** multiscale simulation, coarse-grained, Monte Carlo, fluence, cross-linking

## Abstract

Epoxy resins are critical materials in aerospace applications, yet their mechanical properties, specifically the tensile modulus, can be significantly compromised when exposed to electron irradiation in space environments. To thoroughly examine this degradation, we developed an integrated research approach combining vacuum electron irradiation experiments with multi-scale simulations. Coarse-grained (CG) and Monte Carlo (MC) methods were employed to generate the necessary models and primary knock-on atom (PKA) data, while molecular dynamics (MD) simulations were conducted to model the irradiation and tensile processes. Our findings reveal that the tensile modulus percentage loss of epoxy resin stabilizes as the irradiation dose approaches 1.0×10^1^⁵ eV/cm^2^. The strong agreement between experimental and simulation results validates the accuracy of this methodology. In the epoxy resin systems studied with different degrees of cross-linking, irradiation leads to an increase in the tensile modulus of the low cross-linked structures with a maximum increase of 21.46%, and it leads to a decrease in the tensile modulus of the high cross-linked structures with a maximum decrease of 8.03%. This multi-scale approach has been successfully applied to investigate the trends and causes of tensile modulus changes in epoxy resins after electron irradiation. It can be used to explore the changes in the properties of a wider range of polymers after irradiation.

## 1. Introduction

Epoxy resins are one of the most important thermosetting polymers today because of their unique characteristics and benefits over conventional thermoplastic or thermoset resins [1,2,3,4]. Epoxy resins exhibit minimal shrinkage during the curing process, as well as outstanding mechanical and fatigue strength, chemical resistance, heat resistance, electrical properties, and moisture resistance [3,5,6,7,8,9]. Epoxy resins are widely used in industrial and aerospace applications, for example, to seal electronic devices made of various substrates such as plastic, aluminum, copper, etc., and as protective coatings for structural materials to reduce damage caused by shock and vibration on spacecraft [10,11,12]. However, the normal operation of a spacecraft in a complex and hostile space environment can be affected by its exposure to radiation environments such as high energy particles, plasma, atomic oxygen, and so on [13,14,15]. Among these, particle radiation is considered to be one of the most damaging types of radiation to materials, in particular to sensitive polymers, including epoxy resins [15]. Space particles consist mainly of electrons and protons from cosmic radiation, where high-energy electrons can penetrate the shielding and become embedded in dielectric materials inside spacecraft subsystems. This can affect dielectric materials and thus damage sensitive electronic components [16].

When the epoxy resin is irradiated with electrons, the chain breakage of the resin leads to the formation of free radicals, and the further evolution of the free radicals can lead to chain breakage and cross-linking reactions in the resin system, and thus to changes in the cross-linking network of the resin itself [17,18]. As the epoxy structure changes, the electrical [19], thermal [20,21,22], mechanical [20,22,23,24,25], and outgassing [26] properties of the epoxy resin will also change. While thermal, electrical, and adhesion properties are also important, changes in tensile modulus are directly related to the mechanical strength and durability of the material [27], which is particularly critical when protecting electronic devices and as protective coatings, and has led to a focus on the mechanical properties of epoxy resin [28]. The thermodynamic properties of bisphenol A-type epoxy resins were analyzed as a function of irradiation dose using calorimetry, dynamic mechanical spectroscopic measurements, and single-week compression experiments by Vignoud et al. [22]. In Vignoud’s study, irradiation resulted in a decrease in the elastic modulus of the rubbery region of the epoxy due to the disruption of the cross-linking points. In recent years, the effect of dosage on the thermal and mechanical properties of epoxy polymers was investigated using thermogravimetric analysis, tensile experiments, and dynamic mechanical analysis by Nguyen et al. [20]. In their study, the tensile strength and Young’s modulus of the epoxy polymers increased and then decreased with the application of a 100 kGy electron beam. The elongation at break decreased with increasing irradiation dose. Therefore, it is essential to investigate the impact of electron irradiation on the mechanical properties of epoxy resins. These experiments can quantitatively analyze the effects of electron irradiation on epoxy resins from a macroscopic point of view, but due to the limitations of the characterization tools, it is difficult to closely relate the changes in the mechanical properties of epoxy resins to their structural changes. A single experimental approach does not describe the details of radiation damage and the mechanism of radiation effects on material properties [29]. Molecular dynamics (MD) simulations have proven to be a powerful tool for studying the molecular structure and mechanical properties of cross-linked networks. All-atom simulations typically require a significant computational effort, which limits their applicability to limited time scales [30,31]. Coarse-grained (CG) methods allow access to larger simulation volumes and time scales than atomic methods, and the mapping of atomic degrees of freedom into the cross-linked network allows the calculation of material properties [32,33,34,35]. However, these are not yet capable of describing how the irradiated electrons interact with the epoxy resin in microsimulation.

Radiation damage begins with the interaction of energetic particles with the material, where the energetic particles disrupt the atomic structure to produce primary knock-on atoms (PKAs). Next is the primary damage phase, where PKAs initiate a collision cascade in the material, creating more defects. The interaction of energetic particles with materials can be approximated as a binary collision and the process can be simulated using Monte Carlo (MC) methods [36]. The collision cascade process requires the simulation of the simultaneous motion of all atoms in the damaged region—taking into account the diffusion-limited dynamics and the potential compounding of displaced atoms—and is more amenable to MD methods [37]. To reduce the loss of PKA information in cross-scale simulations, the PKA information obtained from the MC method is passed as input to MD, and the MD simulation is then initiated [38,39]. In a study by Li et al., a multiscale simulation framework that can fully exploit the PKA energy spectrum was developed and successfully applied to the study of irradiation damage in phosphorus-doped n-type silicon materials, where the simulation results were able to agree well with the experimental values [40]. The method is general and can be used for the cross-scale simulation of irradiation damage in epoxy resin materials.

When building molecular models for MD or MC simulations, the amorphous structure of epoxy resins is more difficult to model than crystals due to its complex cross-linking network. Epoxy resins typically have a wide variety of structures due to different resin and hardener choices, each with unique physical properties. Currently, approximately 75% of the world’s epoxy polymers are synthesized using diglycidyl ether of vanillyl alcohol (DGEVA) and bisphenol A (BPA) [41]. While BPA is a potential health hazard, epoxy resins synthesized using dihydroxyaminopropane of vanillyl alcohol (DHAVA) as the curing agent are bio-based epoxy resins, which are now commonly used and offer a higher level of safety [42,43,44,45]. This epoxy can be used in aerospace and its modeling process has now been explored, but its mechanical properties are still of scientific interest. For molecular simulation, the curing reaction of the resin can be described at the MD scale by coupling a series of chemical bonding changes associated with the cross-linking reaction [46,47,48,49,50]. However, this approach consumes a significant amount of computational time for large epoxy resin systems [51]. The coarse-grained method can effectively solve this simulation time when the simulation system is too large of a problem. When considering an all-atom structure with the coarse-grained method, the number of particles must be calculated is substantially reduced, so that the system can take a longer period of time for the simulation of the reaction process, and at the same time more accurately describe the reaction process. In order to determine the cross-linking network structure of epoxy resins reliably, accurately, and efficiently, researchers are developing a CG modeling technique based on MC simulation methods [52,53,54,55]. This method clearly describes the curing reaction process between the epoxy compound and the curing agent [51].

In this work, we report on a multi-scale approach combining simulation and experimentation, which was used to calculate the effect of electron irradiation on the percentage loss of tensile modulus of epoxy resins. We calculated the effect of different injection volumes on the percentage of tensile modulus loss of epoxy resins using vacuum electron irradiation coupled with environmental experiments. To investigate the mechanism of the effect of irradiation with different injection volumes on the properties of epoxy materials, we further analyzed it using micro scale simulation. Firstly, all-atom cross-linked networks of epoxy resins synthesized from DGEVA and DHAVA as monomers with different degrees of cross-linking were constructed using the coarse-grained method. Then, the interaction between electrons and epoxy resin was described based on the MC method, and PKA information was obtained. Then, MD simulation was used to simulate the effect of different injection amounts on the tensile modulus percentage loss of epoxy resin, and the effect of different cross-linking degrees on the tensile modulus loss rate of epoxy resin was further investigated.

## 2. Methods

**Figure 1:**
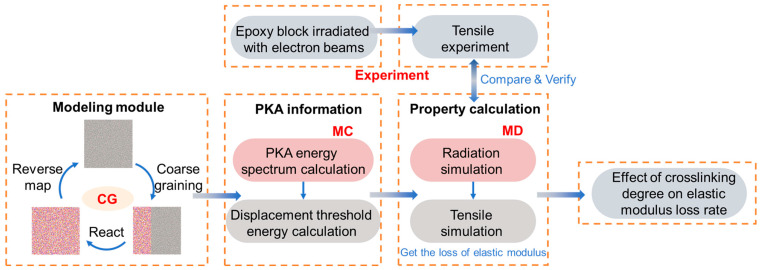
Flow of multiscale simulations and experiments.

Figure 1 illustrates the research approach used to investigate the effect of electron irradiation on the percentage loss of the tensile modulus of epoxy resins. Firstly, we obtained the effect of electron irradiation on the percentage loss of the tensile modulus of epoxy resins at the macroscopic scale by using vacuum electron irradiation-coupled environment experiments. Then, we established the microscopic model of epoxy resin by coarse-graining, calculated the PKA information based on Monte Carlo method, and then calculated the effect of electron irradiation on the percentage loss of the tensile modulus of epoxy resin at the microscopic scale using the molecular dynamics method. The results were confirmed by comparison with the macroscopic results, on the basis of which we further calculated the effect of the cross-linking degree on the percentage loss of the tensile modulus of epoxy resins.

### 2.1. Electron Irradiation Experiment

**Figure 2:**
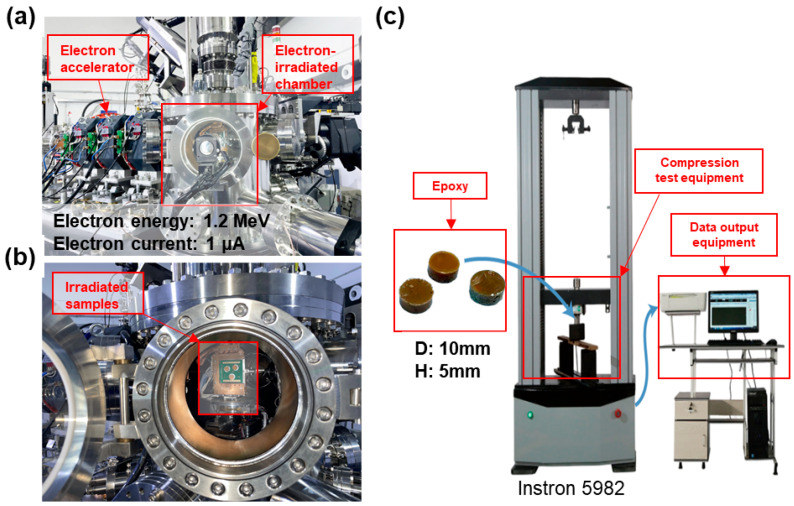
Electron radiation experimental equipment. (**a**) Electron accelerator. (**b**) Electron irradiation chamber. (**c**) Electron universal compressor.

The elastic modulus experiments on epoxy resin colloids under electron beam irradiation were carried out using an electron beam irradiation apparatus, produced by the Shanghai Institute of Applied Physics, Chinese Academy of Sciences. The equipment consists of an electron accelerator and an irradiation chamber connected together, as shown in Figure 2a,b. Energetic electrons are generated by the electron accelerator and the electron energy is obtained by accelerating the electrons with an induced electric field. The epoxy resin sample was fixed to the target stage within the chamber, with electrons produced by the accelerator directed onto the sample. As the electrons can be dissipated by any medium in the atmosphere, a vacuum was applied to the interior of the irradiator.

The electron energy and current were 1.2 MeV and 1 μA [56,57], respectively, which correspond to the equivalent irradiation energy and current of the Jupiter probe. The energy of the electrons produced by the electron accelerator is guaranteed by the use of a thin aluminum film, which can block any electron whose energy is lower than 1.2 MeV. A uniform electron current density is achieved using a current density regulator, based on the electron current density measured by the faraday cup. Hence, the electron radiation equipment can produce a stable target electron radiation. The epoxy sample was mounted onto an irradiation target with an irradiation area of 25 cm^2^. The irradiation equipment was able to achieve an electronic accelerator irradiation uniformity of ≥90, to ensure uniform irradiation of the epoxy resin. The maximum penetration depth of epoxy resin under electron irradiation is 5 mm, and the depth of the test block was 5 mm, which ensures that the test block was thoroughly irradiated. The target was oriented towards the accelerator to ensure that the electrons irradiated the sample perpendicularly. The electron flux was divided into seven sets of injection levels, *ϕ* = 0, 0.1 × 10^15^, 0.4 × 10^15^, 0.7 × 10^15^, 1.0 × 10^15^, 1.5 × 10^15^, and 2.0 × 10^15^ eV/cm^2^.

In order to make the experimental loading method closer to the actual working conditions, the epoxy resin samples were processed in cylindrical blocks 10 mm in diameter and 5 mm in height, with the material properties of each sample being uniform. The irradiated samples were divided into seven groups of three samples each, corresponding to seven irradiation levels. When the target irradiation level was reached for each group, the specimens were removed for epoxy modulus experiments. Epoxy resins are amorphous polymers that are macroscopically isotropic. The tensile and compressive moduli of epoxy resins are very similar at small strains, as shown by the results of Littell et al. [27]. The modulus of elasticity of the epoxy resin at each irradiation level was obtained by averaging the modulus of the samples in each group, and the modulus of each sample was measured three times to take the average value as the experiment modulus. The modulus of the epoxy resin was measured using an electronic universal compression tester (model: Instron 5982, Norwood, MA, USA), as shown in Figure 2c. The temperature during testing the epoxy specimens was 25 °C. In order to reduce the influence of the atmosphere, the samples were stored in a vacuum box after each level of electron fluence experiments and the compression tests were carried out immediately.

### 2.2. Coarse Grained Simulations

**Figure 3:**
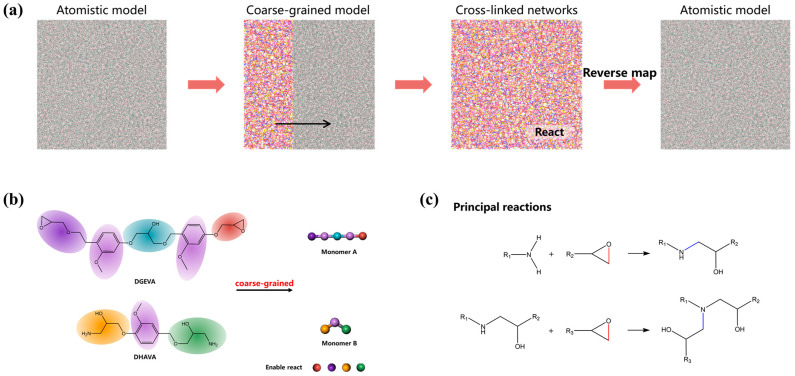
Coarse-grained modeling approach. (**a**) Coarse-grained modeling process. (**b**) Monolithic coarse-graining scheme. (**c**) Main chemical reactions involved.

In order to better characterize the cross-linking network properties of epoxy resins, we used a coarse-grained method to construct the epoxy resin structures. The modeling process is shown in Figure 3a. The simulation of the coarse-granulation process was carried out using the GPU-Accelerated LArge-scale MOlecular Simulation Toolkit (GALAMOST) software package(version 4.0.3) [58,59], and the polymer cross-linking and entanglement numbers were also calculated using the toolkit that comes with the software.

First, the all-atom structure was converted into a coarse-grained model consisting of CG particles based on this coarse-grained method. The key information of the modelled system was retained from the bottom up, while keeping the thermodynamic properties consistent. During the fine-graining process, the basic structural information of functional groups, such as benzene rings, and the bond lengths, bond angles, and dihedral angles between individual coarse grains were preserved. Based on the structural characteristics of DHAVA and DGEVA, we adopted the following coarse-graining scheme: three CG beads were selected to represent the solidifying agent DHAVA, and five CG beads were selected to represent DGEVA [51]. A total of 600 CG beads were prepared for DHAVA and 1200 CG beads for DGEVA. To eliminate the effect of boundaries, periodic boundary conditions were set in all three dimensions.

Then, based on the coarse-grained model and in combination with molecular dynamics simulations, the polymerization reaction method was used to reproduce the curing process of the resin cross-linking reaction and to construct the cross-linking network structure of the epoxy resin. Equivalent actual chemical reaction rates were used based on chemical reaction odds [58]. Among these, the DHAVA and DGEVA monomers were coarse-grained, as shown in Figure 3b, which ensured the integrity of functional groups such as benzene rings and provided as many degrees of freedom of the monomers as possible. As shown in Figure 3c, the reaction between DHAVA and DGEVA takes place in two steps. In the first step, the primary amine undergoes a ring-opening reaction with the epoxy group to produce the hydroxyl group and the secondary amine. In the second step, the already reacted amine can undergo a ring-opening reaction with the epoxy group to form the hydroxyl group [51]. This allows the CG beads at the ends of DHAVA and DGEVA to be reacted twice and once, respectively. In the curing reaction process of the coarse-grained model, the deformation of the bonds between the coarse grains is determined by the reaction probability, which depends on the activation energy barrier of the reaction and the corresponding reaction rate [60]. It is known from experiments that the activation energy of the hardening reaction is approximately *E_a_* = 57.48 kJ mol^−1^ [61]. The activation energy is related to the reaction probability [62], i.e., $P_{r}(t)=A\exp[-E_{a}/(N_{A}K_{B}T)]\frac{\left[M(t)\right]}{\left[M_{0}\right]}$, where *A* is the dimensionless correction factor, *A* = 2.2 × 10^5^, *N_A_* is Avogadro’s number, and *K_B_* is the Boltzmann’s constant. Here, [*M_0_*] denotes the monomer concentration (or number density in the simulations) at the beginning of polymerization, while [*M*(*t*)] is the instantaneous concentration at the specific time t during the polymerization. Therefore, we can obtain the corresponding reaction probability based on *E_a_*, i.e., *P_r_* (0) = 2.49 × 10^−3^. A reaction probability of 2.5 × 10^−3^, close to that value, was used by Wang et al. to simulate the curing reaction and generate the coarse-grained cross-linked network structure of the epoxy resin [51].

Finally, the coarse-grained structure was converted to an all-atom structure of the cross-linked network using a reflectance technique, thus complementing the molecular details on a top-down scale. The process was realized by the GALAMOST software, which enables the replacement of coarse-grained bonding information with the help of simplified molecular input line entry system (SMILES) molecular fingerprints. SMILES strings facilitate the precise description of the structure of an organic substance in a concise form, and complex intermolecular reactions can be realized by simple string substitution. Some of the unreacted DHAVA and DGEVA monomers were removed based on the desired degree of cross-linking. Then, the CG cross-linked network structure was reduced to the all-atom cross-linked network structure according to the correspondence between CG beads and all-atoms. In order to ensure the stability of the structure, we subjected the all-atom structure to relaxation under the NPT system until the volume of the box was almost unchanged. The size of the stabilized box was 134.3 × 128.7 × 79.78 Å^3^. At this point, we obtained the all-atom cross-linked network structure of the epoxy resin.

### 2.3. Monte Carlo Simulations

Energetic electrons produce fast moving atoms called PKAs when they first impact an atom. PKAs are the starting point of collisional cascades within a material, so it is important to obtain and use as much information as possible about the properties of PKAs [40]. In this work, PKA information simulations were performed using the extreme environmental radiation effects technology computer-aided design (ERETCAD, version 1.0), which was developed primarily based on GEANT4 (GEometry ANd Tracking) [63].

We used a physical list called standardNR to simulate the transport of heavy ions in BJTs and the production of PKAs. A single Coulomb scattering process was included in standardNR. This physical list also explicitly integrated the classical equations of motion for scattering events, resulting in precise tracking of both the projectile and the recoil target nucleus [64,65]. The MC method allows for the characterization of the particle–target interaction, and thus obtains information on the PKA generated by electrons in the epoxy resin material, which can be applied to subsequent MD simulations. In this paper, the incident electron energy chosen for the MC simulations was 1.2 MeV, which is consistent with the experiments, and a total of 10 electrons were incident to ensure the convergence of the results. After the calculation, the PKA information in the structure was counted. It was found that the PKA atoms were mainly C, N, and O, that the H PKA energy was very low, and that the number of H PKA was small. Therefore, the subsequent MD simulations will be mainly based on these three PKAs.

### 2.4. Molecular Dynamics Simulations

After constructing an all-atom cross-linked network model and obtaining the PKA energy spectra, the MD method was used to simulate the changes in the tensile modulus percentage loss of the epoxy resin after electron irradiation at different injection levels and cross-linking degrees. MD calculations can accurately obtain the changes in the molecular structure of the epoxy resin system during irradiation and stretching. The MD calculations in this paper were performed using the LAMMPS code (29 September 2021—Update 3) [66]. The simulations were carried out using a ReaxFF force field [67] containing four elements, C, O, N, and H, to minimize the energy of the coarse-grained model and relaxing the NPT ensemble at a temperature of 300 K until the structure was stable. The time step of the simulation was set to 0.1 fs.

After preparing the initial structure, kinetic energy was supplied to the PKA at the same injection rate as in the experiment. We extracted the energies and numbers of different PKAs at each injection from the PKA energy spectra and removed PKA atoms with energies less than the displacement threshold energy to accelerate the irradiation efficiency. To maintain the relative stability of the system, only five randomly selected PKA atoms were irradiated with kinetic energy at a time, and the system was allowed to return to steady state for the energy loading of the next batch of PKA atoms during a 1 picosecond (ps) relaxation process. We found that the system energy was already stabilized within the 1 ps relaxation time, so it was not necessary to irradiate the system for a few seconds as in the macroscopic experiments. We then irradiated the epoxy resin structures with different degrees of cross-linking with 0.1 × 10^15^ eV/cm^2^ injections, to subsequently investigate the effect of different degrees of cross-linking on the tensile modulus percentage loss of the epoxy resin.

After irradiating the epoxy resin, we stretched the damaged structure at a rate of 0.02% strain per ps, calculated the tensile modulus within 0.5% strain, and calculated the tensile modulus percentage loss for each structure using the tensile modulus of the undamaged structure as a reference.

## 3. Results and Discussion

### 3.1. Validation of Epoxy Model

**Figure 4:**
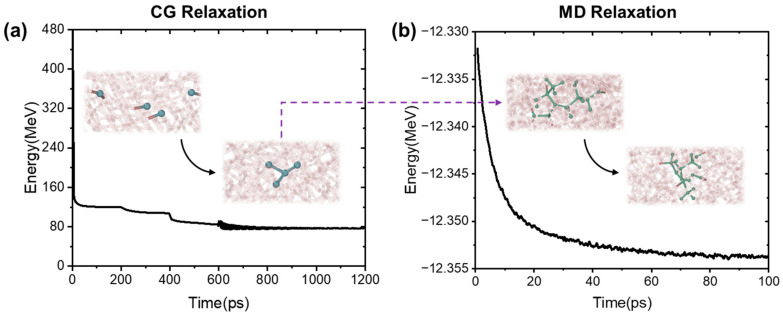
Epoxy model relaxation. (**a**) CG relaxation. (**b**) MD relaxation.

Figure 4a shows the energy changes during coarse-graining. The initial structure consists of monomer molecules without cross-linking, and under prolonged relaxation based on the reaction probability, new chemical bonds are generated between some of the monomers. The overall energy of the process decreases and has small fluctuations around the steady state energy due to the gradual stabilization of the structure. This fully reflects the stability of the structure of the CG process and the validity of the resulting structure. The end of the CG process means that the reaction process has been completed, and the material has been converted to an all-atom structure by a fine-grained process. Since the force field in the MD uses the ReaxFF reaction force field and the finely grained structure was not a stable structure, further relaxation was required at the MD scale to obtain a steady-state all-atom structure. Since the ReaxFF force field was very accurate in describing intermolecular reactions, the cross-linked chains were not disrupted during relaxation. As the relaxation proceeds, the energy of the system gradually decreased and became stable, resulting in a stable all-atom structure. This process illustrates the stability of the structure of the MD process and the fact that each model is a stable structure at the beginning of irradiation.

### 3.2. PKA Information

**Figure 5:**
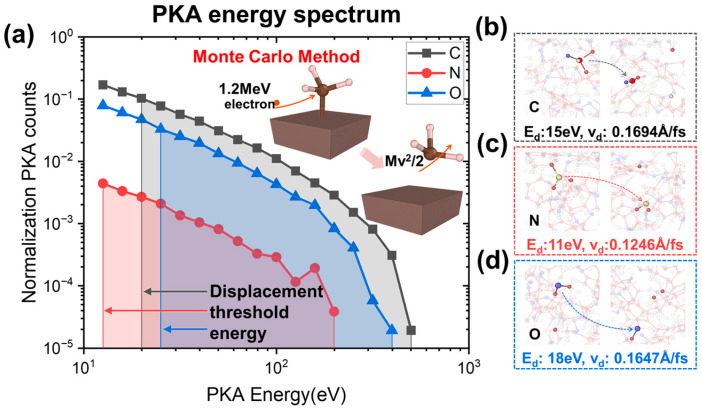
PKA Information. (**a**) PKA energy spectrum. (**b**–**d**) Displacement threshold energy of C, N, and O atoms.

The PKA energy spectrum of the epoxy resin can be calculated using the Monte Carlo method as shown in Figure 5a, which demonstrates the relationship between the normalized PKA number of the main atoms (CNO atoms) and the PKA energy, where the C atoms are the most numerous and have the widest energy range. The off-site domain energies of the C, N, and O atoms can then be calculated, as shown in Figure 5b–d, with off-site domain energies of 15 eV, 11 eV, and 18 eV, respectively.

### 3.3. Irradiation Process

**Figure 6:**
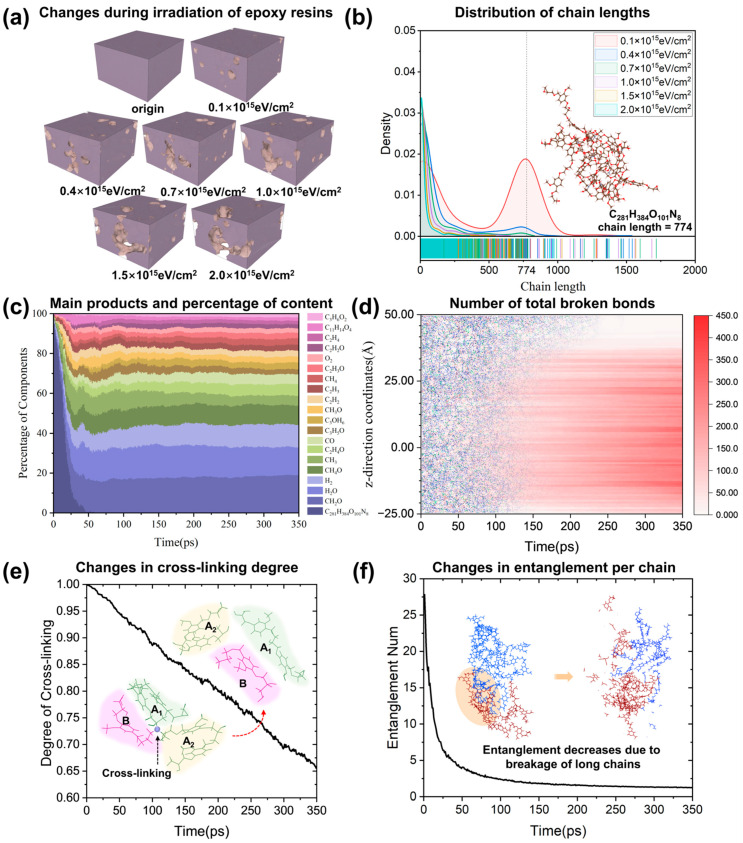
Molecular information characterization of irradiation processes. (**a**) Molecular void changes. (**b**) Chain length distribution changes. (**c**) Major product content. (**d**) Total number of broken bonds in the *z*-direction. (**e**) Changes in the number of cross-linking sites. (**f**) Change in the number of entanglements per chain.

A molecular dynamics approach was used to simulate the irradiation process of the epoxy resins at different injection volumes. The horizontal axis of the five plots in Figure 6 shows the irradiation time, while the description of the irradiation process in Section 2.4 above shows that the PKA particles were batched and relaxed for 1 ps between batches to ensure that the structure reached steady state. Therefore, the change in structure with irradiation time is consistent with the change in structure with injection volume. Figure 6a shows the initial structure as well as the structure after irradiation at each injection volume. In order to observe the damage to the epoxy resin by electron irradiation more clearly, the surface of the epoxy resin structure was visualized using the open visualization tool (OVITO, version 3.10.6) [68], which allows the increase and expansion of the pores to be observed more clearly. Figure 6b shows the chain length statistics of the structure, among which the C_281_H_384_O_101_N_8_ [(C_25_H_32_O_9_)_9_ (C_14_H_24_O_5_N_2_)_4_] molecule consisting of 9 DGEVA molecules and 4 DHAVA molecules had the largest quantity at initial irradiation. The macromolecules in the structure are decomposed into small molecules in about 50 ns. Such a fast decomposition rate results in a structure dominated by macromolecules after irradiation with only 0.1 × 10^15^ eV/cm^2^ injections, and a structure dominated by small molecules after irradiation with higher injections. As the amount of injection increases, the long chain gradually decreases, and the short chain begins to increase. This indicates that chain scission reactions occur during irradiation. It is noteworthy that some longer chains can be seen at high injection levels, suggesting the presence of a small number of cross-linking reactions leading to chain growth. The phenomenon is consistent with that observed experimentally by Nguyen et al. [20].

To further observe whether the post-reaction chain breakage affects the basic structure of the epoxy resin, the molecular composition was counted at various times during the irradiation process and some of the most abundant defect-containing chains, small molecules, and free radicals were plotted, as can be seen in Figure 6c. The three most abundant small molecules produced are CH_2_O, H_2_O, and H_2_. However, as most of the small molecules produced are of low molecular weight, they do not have a large effect on the overall properties. The formation of small molecules means that the chains of the epoxy resin are broken, and these breaks occur either in the side chains or lead to breaks in the main chain, thus affecting the mechanical properties of the epoxy resin. In order to observe whether large damage occurs locally during the irradiation process, the total number of broken bonds along the *z*-direction of each layer was counted with the change in irradiation time, as shown in Figure 6d. The distribution of broken bonds along the z-direction is uniform.

To further analyze the changes in the structure of the epoxy resin during irradiation, the changes in cross-link density and the number of single chain entanglements were counted with irradiation time, as shown in Figure 6e,f. The cross-link density gradually decreases in an almost linear trend from 100 percent to 65 percent. A higher cross-link density tends to indicate a more brittle structure, and usually the breakage of a cross-link point results in the breakage of a long cross-linked chain into three parts, which leads to better flowability, i.e., the resin is less stiff and more plastic. Analysis of the structure shows that the decrease in entanglement is mainly due to the breaking of long chains. The decrease in entanglement is faster than the decrease in cross-linking points, basically because amorphous molecular chains are mobile, which causes the points of entanglement to disappear as the molecular chain moves. However, the decrease in cross-linking points can also be due to the breaking of the bonds in the region, which usually requires a large amount of energy. The higher the entanglement number, the better the mechanical properties of the resin in the elastic region under load.

### 3.4. Tensile Simulation Results

**Figure 7:**
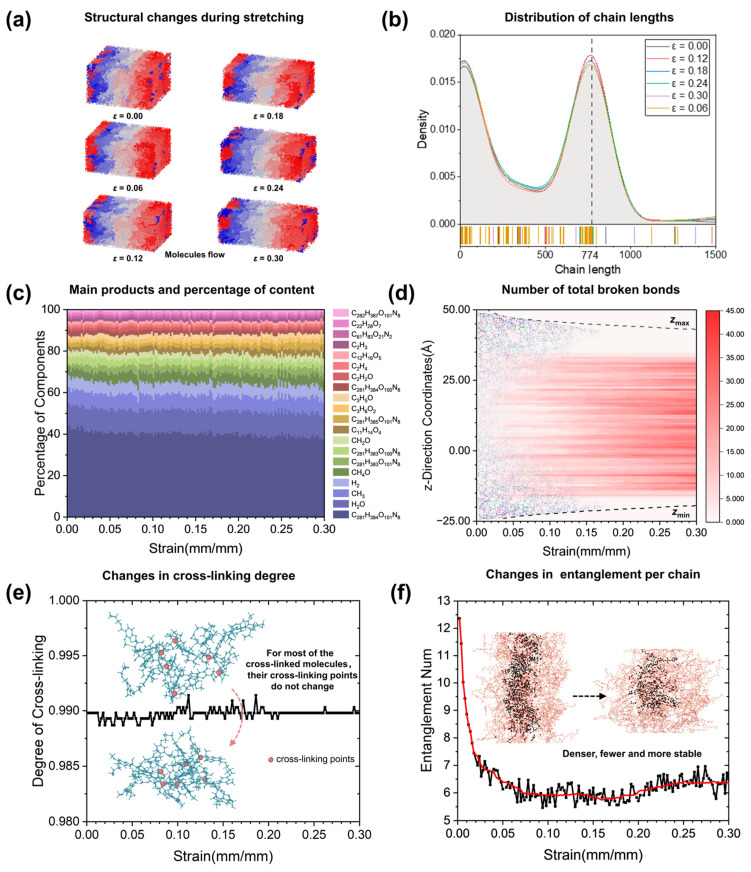
Molecular information characterization of the stretching process. (**a**) Structural changes. (**b**) Chain length distribution changes. (**c**) Major product content. (**d**) Total number of broken bonds in the *z*-direction. (**e**) Changes in the number of cross-linking sites. (**f**) Changes in the number of entanglements per chain.

A molecular dynamics method was used to simulate the stretching process of the epoxy resin at different injection amounts. Figure 7a shows the initial structure and its changes in the position of each molecule during stretching up to a strain of 0.3. As the stretching progresses, the length in the stretching direction increases while the lengths in the other two directions decrease. To accommodate the change in the shape of the box, the molecules inside appear to flow. Figure 7b shows the chain length statistics of the structure. The peak length and structure of the chain lengths are the same as in Figure 6b. As the stretching progresses, it can be seen that the chain length distribution remains almost unchanged, indicating that there is very little bond breaking in the system.

To further observe whether molecular breakage and reorganization occurs during stretching, we counted the molecular composition of the system during stretching, and some of the highest contents of defect-containing chains, small molecules, and free radicals can be seen in Figure 7c. The small molecules here are mainly produced by the irradiation process of the pre-sequence, and the number of different molecules hardly changes during the stretching process. When combined with the statistical curve of the total number of broken bonds in the z-direction (Figure 7d), it can be seen that the total number of broken bonds in the stretching process is much smaller than that in the irradiation process, and the molecular composition of the system is almost unchanged.

The changes in cross-link density and number of entanglements during stretching were also counted, as shown in Figure 7e,f. The degree of cross-linking remains almost unchanged, and most of the cross-linking points only change their positions and did not change the cross-linked molecular structure. Systems with high cross-linking are able to maintain their degree of cross-linking during stretching, and systems with low cross-linking are easier to maintain. However, the number of entanglements still gradually decreases and finally remains close to 6.0, and still had a tendency to increase. By observing the structure, it was found that the originally dispersed molecules with entanglements became more tightly entangled during the stretching process, causing the number of entanglement points to converge on a value, as shown by the fitted curve in red in Figure 7f. During the subsequent stretching, the molecules around the entangled points appear to have new entanglement interactions with the entangled macromolecules as they flow, but the appearance of such interactions is less likely and transient. For irradiated epoxies, the number of entanglements is already very low, but according to the analysis, stretching leads to a further reduction in the number of entanglements.

For epoxy resins that can be designed on molecular optoelectronic devices, their optical properties are also of interest. Therefore, in addition to the structural characteristics described above, the effect of stretching on the refractive index (RI) of the structure after irradiation with different injections was calculated. The RI was obtained from the open square of the dielectric constant of the structure, which was calculated as follows: [69,70,71]

(1) $$\varepsilon =1+\frac{\left(\langle M^{2}\rangle -{\langle M\rangle }^{2}\right)}{3Vk_{B}T\varepsilon_{0}}$$

(2) $$n=\sqrt{\varepsilon }$$

where, **M** is the dipole moment of the system at one-time step, $\langle M^{2}\rangle$ is the average value of the dipole moment squared, ${\langle M\rangle }^{2}$ is the square of the mean dipole moment values, *V* is the volume, *k_B_* is the Boltzmann constant, *T* is the temperature, *ε*_0_ is the vacuum dielectric constant, and n is the refractive index.

As illustrated in Table 1, the calculated alterations in the refractive index of the epoxy resin have been demonstrated to occur both prior to and following the process of stretching, subsequent to the application of diverse injections. The square of the refractive index of the epoxy resin is defined as the dielectric constant. The resultant dielectric constant of the unirradiated epoxy resin has been calculated to be 3.583, a value that falls within the range of experimental values reported (3~4) [72]. The data obtained for the calculated refractive index demonstrate a decrease in the epoxy resin refractive index following both irradiation and stretching. The decrease in polarizability within the resin is attributed to chain-breaking reactions occurring during irradiation and a reduction in intermolecular interactions during stretching. This, in turn, results in a decrease in the dipole moment, which consequently leads to a decrease in the refractive index. Consequently, when utilizing resins in optical devices, it is imperative to meticulously engineer them to mitigate the detrimental effects of both irradiation and external loads.

### 3.5. Comparison of Tensile Results

First, we experimentally determined the effect of different injection volumes on the rate of loss of the tensile modulus of epoxy resin. The epoxy resin modulus experiments were carried out under electron irradiation, and after each set of irradiation experiments, the corresponding set of samples was removed for the elastic modulus experiments. To minimize the effect of atmospheric conditions on the measurements, the irradiated specimens from each group were stored in vacuum boxes and immediately subjected to compression modulus experiments. Through the sealed storage, to ensure that the measurement results of epoxy resin samples under electronic irradiation were reliable, by collating the elastic modulus of epoxy resin samples from each group of the irradiation experiments, the effect of different irradiation doses on the elastic modulus of epoxy resin was determined. From the figure, it can be seen that the modulus change of the epoxy resin sample increases rapidly with the increase in injection volume, and gradually tends to be stable after the injection volume reaches *ϕ* = 1.0 × 10^15^ e/cm^2^. It is assumed that the molecular chain of the epoxy resin breaks rapidly during the initial stage of irradiation and the modulus decreases. After the cumulative degradation from irradiation injection, the structure of the epoxy resin with small molecular chains becomes more stable, and the modulus tends to be stable. Considering the fact that the modulus of the epoxy resin under electron irradiation only becomes significant when the amount of injection accumulates to a certain degree, the importance of the cumulative effect of irradiation time is confirmed, which supports the necessity of carrying out the experiment of epoxy resin irradiation with the consideration of long-term cumulative effects for deep space exploration.

**Figure 8:**
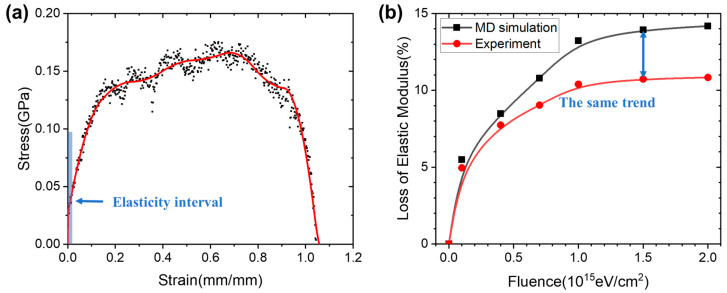
The percentage of tensile modulus loss varies with injection volume. (**a**) Tensile curves of unirradiated epoxy resins. (**b**) Comparison of experimental and simulation results.

To cross-check with the experimental results, we used the established irradiated model in Section 3.3 to stretch at the molecular level using the MD method and calculated the tensile modulus percentage loss of the epoxy resin at different injection levels. The all-atom model of epoxy resins at different injection levels was irradiated and then stretched. Figure 8a shows the stress–strain curve of unirradiated epoxy resin during stretching, and the data corresponding to 0.5% strain in the elastic region were selected and fitted to obtain the value of the elastic modulus of this material. As epoxy resins belong to the amorphous body system, their strain process will not be as stable as that of crystalline materials and low frequency oscillations will often occur. However, due to the low strain rate used during the simulation, most of the oscillations occur in the plastic phase, which does not affect the value of the Young’s modulus calculated in the elastic region. On this basis, the effect of irradiation on the percentage loss of elastic modulus was calculated by calculating the value of elastic modulus before and after irradiation of the epoxy resin at each injection amount, as shown in Figure 8b. Meanwhile, the microhardness of the epoxy resin before and after irradiation was calculated using the approach of Zeng et al. [73]. The percentage loss was also counted in Table 2. As the irradiation dose increased, there was a continuous decrease in the hardness of the material due to the weakening of the intermolecular interactions of the epoxy resin. The microhardness calculations were carried out in such a way as to be consistent with the change in the modulus of elasticity. By comparing with the experimental results, it is easy to find that the effect of irradiation on the loss rate of elastic modulus in the simulation is consistent with the experimental results in terms of its trend. As the epoxy blocks in the experiment were not as homogeneous and pure as the material in the simulation, there is still a slight discrepancy in the calculated values.

### 3.6. Epoxy Resins with Different Cross-Linking Degrees

When investigating the effect of different injection volumes on the tensile properties of epoxy resins, the decrease in cross-linking degree was more obvious with the increase in injection volume, and the decrease in cross-linking degree tended to affect the mechanical properties of epoxy resins [74]. For example, in the study by Shokuhfar et al., an 81% cross-linked epoxy showed a 37.41% increase in modulus compared to 0% epoxy [75]. So, it is necessary to pay attention to whether the cross-linking degree itself affects the percentage loss of modulus of elasticity.

**Figure 9:**
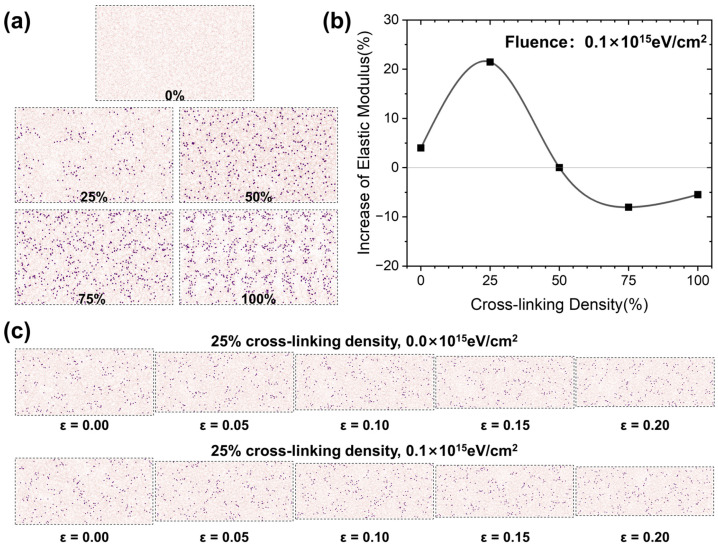
Effect of cross-link density on the percentage loss of tensile modulus. (**a**) Structures of epoxy resins at various degrees of cross-linking. (**b**) Variation of tensile modulus increase rate with cross-link density. (**c**) Snapshots of the tensile process before and after irradiation of 25% cross-linking degree epoxy resin.

Since the rate of loss of Young’s modulus is not sufficiently intuitive, the percentage increase of Young’s modulus has been used as the vertical axis to describe the effect of radiation on the rate of loss of Young’s modulus. As shown in Figure 9a, we constructed epoxy resin models with different degrees of cross-linking. This is because in the previous section’s simulation, we found that the cross-linking degree of the epoxy resin system changes relatively little in the set of models with an irradiation dose of 0.1 × 10^15^ eV/cm^2^. Therefore, the irradiation process can be reduced to influence the cross-linking degree at this injection amount. This made it easier for us to investigate the effect of different cross-linking degrees on the percentage loss of the elastic modulus of the epoxy resin. As shown in Figure 9b, the elastic modulus of epoxy resin at a 0–50% cross-linking degree was increased after irradiation. Epoxy resins with less than 50% cross-linking were suitable for components subjected to lower forces, as they maintained their mechanical properties even after exposure to radiation. Cross-linking and chain breaking reactions occurred mainly during the irradiation process. The elastic modulus of the 0% cross-linked resin was increased due to a small number of cross-linking reactions, while the elastic modulus of the 100% cross-linked epoxy resin was decreased due to a small number of bond breaks after irradiation. As shown in Figure 9c, the number of cross-links in the structure before and after irradiation at a 25% cross-linking degree remained almost unchanged during stretching, but the cross-link points were moving in sync with the polymer chains during stretching. For systems with different degrees of cross-linking, we calculated the free volume of the structure using the construct surface mesh function of OVITO. A 25% cross-linking degree of the resin had a maximum free volume of 3.13% in the structure after relaxation. Therefore, in the process of irradiation, with the occurrence of molecular chain-breaking reactions, the resin molecules continuously adjust their conformation, making the structure more compact and better quality, which in turn improves the elastic modulus of the epoxy resin.

## 4. Conclusions

In this study, a multiscale approach combining both simulation and experimentation was employed to investigate the impact of varying irradiation doses on the tensile modulus percentage loss of epoxy resins, as well as the influence of different cross-linking degrees on this loss rate. The main contribution of this paper is the development of a comprehensive multiscale research methodology that integrates vacuum electron irradiation coupled with environmental experiments and microscale simulations. The simulations utilized the MD method, with models constructed using the CG method, and PKA information was derived from the MC method. The CG method significantly enhanced the efficiency of molecular simulations while maintaining an accurate depiction of the monomer reaction processes. The PKA energy spectra obtained through the MC method effectively transferred irradiation data to the MD simulations.

Using this multiscale simulation approach, the irradiation process of epoxy resins under six different injection doses and the subsequent stretching behaviors of the irradiated structures were simulated. To explore the relationship between tensile modulus loss and structural properties, factors such as chain length distribution, composition, cross-linking degree, and entanglement count were analyzed throughout the simulation. During irradiation, the molecular chains transitioned from a long-chain-dominated structure to one dominated by shorter chains, producing small molecules such as CH_2_O, H_2_O, and H_2_. Simultaneously, the degree of cross-linking and the number of entanglements gradually decreased. During stretching, while the molecular chains and cross-linking degree showed minimal change, the number of entanglements further decreased due to relative sliding between the chains. The irradiation-induced loss of tensile modulus was evaluated by analyzing the stretching results, which showed a trend consistent with experimental data. Initially, as the irradiation dose increased from 1.0 × 10^15^ eV/cm^2^, the percentage loss of elastic modulus gradually rose, eventually stabilizing at approximately 10.5%.

To investigate the effect of cross-linking degree on the tensile modulus loss, models with varying cross-linking degrees were created and subjected to the same irradiation and stretching conditions. Simulation results revealed that epoxy resins with less than 50% cross-linking exhibited an improvement in elastic modulus after irradiation, while those with more than 50% cross-linking experienced a decrease in modulus. Notably, the epoxy resin with a 25% cross-linking degree demonstrated the highest modulus enhancement, approximately 21.46%, following irradiation.

The successful integration of simulation and experimentation has proven effective in studying the effect of electron irradiation on the tensile modulus loss rate of epoxy resins. This approach can also be extended to investigate the influence of irradiation on the properties of polymer materials.

## Figures and Tables

**Table 1:** The effects of stretching on the refractive index of the epoxy resins irradiated with different injections.

Injection (10^15^ eV/cm^2^)	RI Before Stretching	RI After Stretching
0	1.893	1.700
0.1	1.767	1.660
0.4	1.756	1.659
0.7	1.716	1.691
1.0	1.692	1.619
1.5	1.732	1.716
2.0	1.622	1.668

**Table 2:** Percentage loss of the microhardness and elastic modulus of epoxy resins after irradiation.

Injection (10^15^ eV/cm^2^)	Percentage Loss of the Microhardness	Percentage Loss of the Elastic Modulus
0.1	5.490	0.1498
0.4	8.480	0.1585
0.7	10.78	0.2024
1.0	13.23	0.2195
1.5	13.93	0.2368
2.0	14.18	0.3477

## Data Availability

The original contributions presented in this study are included in the article. Further inquiries can be directed to the corresponding authors.
